# Origin and global diversification patterns of tropical rain forests: inferences from a complete genus-level phylogeny of palms

**DOI:** 10.1186/1741-7007-9-44

**Published:** 2011-06-16

**Authors:** Thomas LP Couvreur, Félix Forest, William J Baker

**Affiliations:** 1 The New York Botanical Garden, 200th Street and Kazimiroff Boulevard, Bronx, NY 10458-5126, USA; 2 Royal Botanic Gardens, Kew, Richmond, Surrey, TW9 3AB, UK; 3 Institut de Recherche pour le Développement (IRD), UMR DIA-DE, DYNADIV researche group, 911, avenue Agropolis, BP 64501, F-34394 Montpellier cedex 5, France

## Abstract

**Background:**

Understanding how biodiversity is shaped through time is a fundamental question in biology. Even though tropical rain forests (TRF) represent the most diverse terrestrial biomes on the planet, the timing, location and mechanisms of their diversification remain poorly understood. Molecular phylogenies are valuable tools for exploring these issues, but to date most studies have focused only on recent time scales, which minimises their explanatory potential. In order to provide a long-term view of TRF diversification, we constructed the first complete genus-level dated phylogeny of a largely TRF-restricted plant family with a known history dating back to the Cretaceous. Palms (Arecaceae/Palmae) are one of the most characteristic and ecologically important components of TRF worldwide, and represent a model group for the investigation of TRF evolution.

**Results:**

We provide evidence that diversification of extant lineages of palms started during the mid-Cretaceous period about 100 million years ago. Ancestral biome and area reconstructions for the whole family strongly support the hypothesis that palms diversified in a TRF-like environment at northern latitudes. Finally, our results suggest that palms conform to a constant diversification model (the 'museum' model or Yule process), at least until the Neogene, with no evidence for any change in diversification rates even through the Cretaceous/Paleogene mass extinction event.

**Conclusions:**

Because palms are restricted to TRF and assuming biome conservatism over time, our results suggest the presence of a TRF-like biome in the mid-Cretaceous period of Laurasia, consistent with controversial fossil evidence of the earliest TRF. Throughout its history, the TRF biome is thought to have been highly dynamic and to have fluctuated greatly in extent, but it has persisted even during climatically unfavourable periods. This may have allowed old lineages to survive and contribute to the steady accumulation of diversity over time. In contrast to other plant studies, our results suggest that ancient and steady evolutionary processes dating back to the mid-Cretaceous period can contribute, at least in part, to present day species richness in TRF.

## Background

Tropical rain forests (TRF) are the most biodiverse terrestrial ecosystems on the planet [1]. They are characterised by a closed, multilayered canopy dominated by flowering plants (angiosperms [1]) and occur only in frost-free areas with high mean monthly temperatures and precipitation, and low seasonality [2]. Today, TRF covers just 7% of the Earth's surface [3] in equatorial zones of the Americas, Africa and the Indo-Pacific, and is highly threatened by human activity [4]. The origin and evolution of species-rich biomes raises fundamental questions in evolutionary biology [5] and, as such, the diversification of TRF has been much debated [3,6].

Even though it is generally agreed that TRF is a relatively old biome, the location and timing of its origin remain uncertain mainly because the fossil record for tropical regions is highly incomplete, especially during the Cretaceous [7,8]. Some direct [9] and indirect [10] evidence suggests that TRF was present in the mid-Cretaceous period (100 million years ago (Ma)) at middle paleolatitudes (for example, Laurasia) while other studies indicate that the first paleoflora attributable to modern day TRF are found in the Early Paleocene of North America [11] and Late Paleocene of South America [7,8,12] and Africa [13-15].

**Figure 1:**
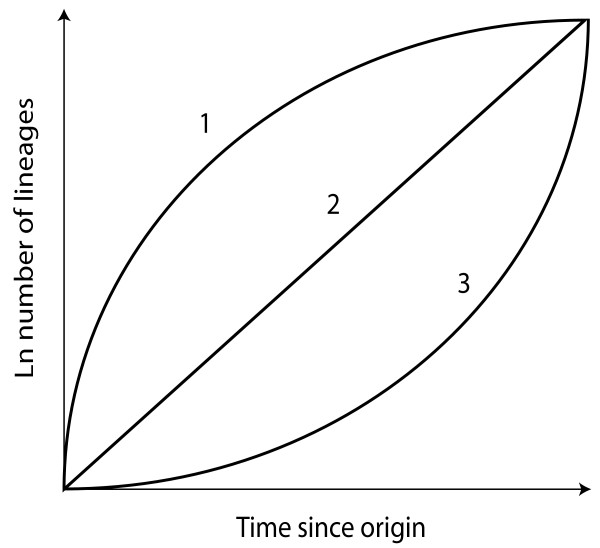
**Lineage-through-time (LTT) plots for three alternative hypothetical diversification models of tropical rain forest (TRF) evolution**. (1) Decrease in diversification rates since origin with early radiation; 'ancient cradle model'; (2) constant diversification rate, 'museum model'; (3) increase in diversification rates since origin with recent radiation; 'recent cradle model'.

Whereas previous views suggested that the TRF biome has been ecologically stable over long periods of time [16], more recent data indicate that it is highly dynamic [17] having fluctuated both in extent [14,18] and in the diversity of plants that it sustains [7,8]. These views have led to three general evolutionary hypotheses that explain the high levels of present day species diversity found within TRF: (i) early, rapid speciation in response to favourable climatic conditions followed by a deceleration of diversification rates due to global cooling [19] and contraction of TRF (referred to here as the 'ancient cradle model', see [10,20,21]); (ii) constant diversification rates coupled with low extinction rates leading to a gradual accumulation of lineages in response to a long-lasting and stable tropical ecosystem (the 'museum model', see [16,22]); and (iii) an increase in diversification rates towards the present in response to climatic, tectonic or biotic changes (the 'recent cradle model', see [23-25]). These hypothetical processes result in alternative lineage accumulation through time and thus different patterns of inferred branch length distributions (Figure 1). Several phylogenetic studies of TRF plant groups have provided evidence in support of the recent cradle model of diversification [25-27]. However, these studies were restricted to low taxonomic levels (species) and thus do not enhance our understanding of how these hypotheses might apply throughout the entire history of TRF, for example, on the long-term diversification dynamics of TRF. In contrast, studies of the early diversification of TRF plant lineages that permit tests of the above hypotheses are rare.

**Figure 2:**
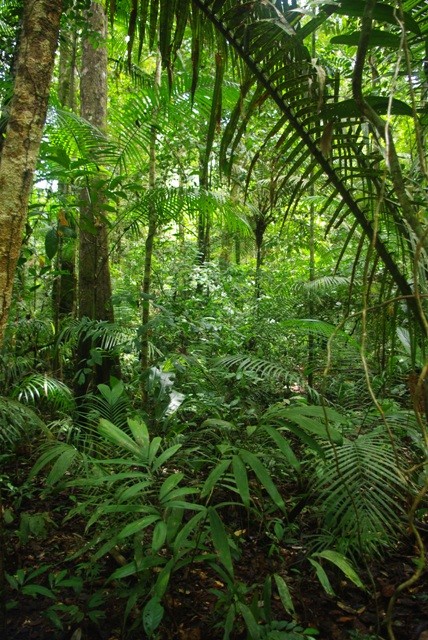
**Example of an understory lowland tropical rain forest in the Parque National do Amazonia (near Itaituba, Pará state, Brazil) dominated by palms**. Foreground *Bactris acanthocarpa *var. *exscapa*, upper right corner *Attalea *sp., middle left *Euterpe precatoria*, background: *Astrocaryum gynacanthum*. Photo: TLPC.

In the absence of a complete fossil record for tropical rain forests, family-level diversification analyses of large pantropical angiosperm groups that are ecologically characteristic of TRF can provide important insights into the historical construction of the biome [5]. In this respect, the pantropical palm family (Arecaceae/Palmae) presents an ideal study group. First, palms are among the most important and characteristic components of TRF ecosystems worldwide in terms of species diversity (approximately 2,400 species), abundance of individuals and impact on the environment [28-30]. Based on the excellent taxonomic knowledge for this family [28], we calculated that over 90% of its species diversity is restricted to TRF (Figure 2). Water and energy-related variables are strong determinants of palm diversity [31,32] and fundamental anatomical constraints inhibit palms from colonising cold environments [33,34]. Second, the known history of palms extends far back into the Cretaceous although the details of the spatiotemporal origin of the family remain controversial [28]. Direct evidence from unambiguous fossils associated with palms suggest that the family was already present during the Turonian (89-93.5 Ma, [35-37]) while more doubtful fossils have been recorded since the Aptian (112 Ma, [28]). More recently, several molecular clock estimates based on angiosperm wide phylogenies suggested a stem age for the family ranging from 91 to 120 Ma [38-41]. These studies were based on a very limited sampling within the family and thus do not provide reliable approximations for the crown node age and early diversification history. To date, most estimates of palm ages have focused on subfamily [42,43] or tribal levels [44-46].

Here we investigate the origin and diversification of palms in space and time using the first complete generic-level sampling for any important TRF-restricted plant family [47]. We estimated speciation events under a Bayesian framework using a relaxed molecular clock approach (BEAST, [48]), while the spatial origin of the family was inferred under a maximum likelihood method that implements the dispersal-extinction-cladogenesis model [49,50]. Finally, we use palms as a model to explore the evolution of TRF biodiversity by testing which of the three TRF diversification hypotheses outlined above corresponds to the diversification history of the family.

## Results and discussion

### Evolutionary origin of palms

**Figure 3:**
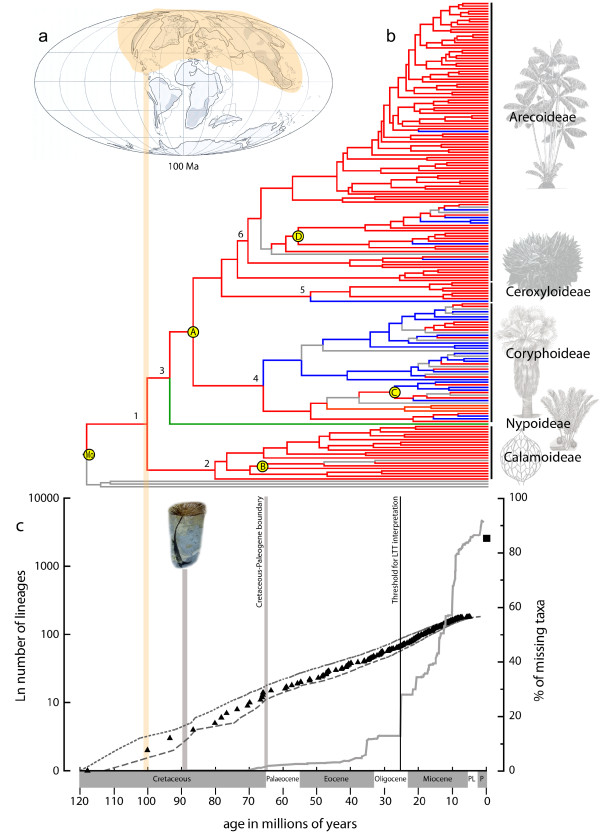
**Area, tempo and mode of palm diversification**. **(a) **Paleomap representing the distribution of landmasses in the mid-Cretaceous period, dark grey upland land, light grey lowland (100 million years (Ma), adapted from Beerling and Woodward [60]). Laurasia, which is the most likely ancestral area reconstructed for the crown node of palms, is highlighted. **(b) **Chronogram showing the three different biomes assigned to each genus. Red: tropical rain forest; green: mangrove; blue: not tropical rain forest; grey: ambiguous. Yellow circles indicate fossil calibration points. The vertical black lines highlight the five subfamilies of palms with an illustration (drawings by Marion Ruff Sheehan, L.H. Bailey Hortorium, Cornell University, except top one (Arecoideae), which is reproduced with permission from Springer from Kahn and de Granville [30]. **(c) **Semilogarithmic mean lineage-through-time (LTT) plot averaged over 1,000 posterior trees from the Bayesian analysis (left axis, triangles) and percentage of missing taxa as a function of time (right axis, grey line). Short dashed line = upper 95% confidence interval; long dashed line = lower 95% confidence interval; filled square = extant number of palms species. Vertical black line indicates threshold up to which the LTT plot is considered reliable even under incomplete taxon sampling. Palm fossil indicates time of earliest known unequivocal fossil for the family (*Sabalites *fossil leaf image reproduced by permission of the Board of Trustees, National Museums Liverpool, Liverpool, UK).

The fossil-calibrated molecular dating of a complete genus-level palm supertree [47] provides for the first time minimum age estimations for all major groups of the palm family (Table 1). The resulting chronogram (Figure 3b) suggests that the diversification of extant lineages of palms started in the mid-Cretaceous period at the Albian-Cenomanian boundary (crown node: 100 Ma, 95% highest posterior density (HPD) 108-92 Ma). The Cretaceous represents an important period for plant evolution as it witnessed the rise and diversification of flowering plants [51,52]. The fossil record indicates that from the Albian to the middle Cenomanian angiosperms diversified extensively, becoming more abundant relative to other plants and establishing themselves as a major part of paleofloras by the end of that period [53]. Our maximum likelihood analysis of geographic range evolution indicates that the most likely distribution of the most recent common ancestor of palms was centred on present day Central/North America and Eurasia, which corresponds to the Laurasian landmass at that time (Figure 3a). Notably, the oldest reliable palm fossils (Turonian to Campanian) have all been discovered in Europe and North America [28]. A Laurasian origin for palms was previously suggested by Uhl and Dransfield [54] based on the prevalence of putatively primitive lineages in the northern hemisphere. From this ancestral area several subsequent dispersal events are inferred into the equatorial regions of South America, Africa and South East Asia, the present day distribution of palms. Finally, our ancestral biome analysis suggests that the earliest palm lineages were restricted to TRF (*P *= 0.984; Figure 3b and Table 1), a result that was further supported by a test of phylogenetic signal of the biome. In fact, adaptation to non-TRF biomes did not arise until the Paleocene within the fan palm subfamily Coryphoideae. Thus, our results support the notion that palms originated in a TRF-like biome and started to diversify during the mid-Cretaceous period in Laurasia.

**Table 1:** Mean estimated ages, 95% confidence intervals and ancestral ecologies for the family and subfamilies

Clade	Age in Ma	95% HPD	Proportional likelihoods of ancestral ecology of branch leading to node^a^
Arecaceae, crown	100.1	92-108.7	**0 = 0.984**/1 = 0.0/2 = 0.0

Calamoideae, crown	80.2	70.3-90.3	**0 = 0.999**/1 = 0.0/2 = 0.0

Nypoideae, stem	93.5	87.5-100.6	**0 = 0.982**/1 = 0.0/2 = 0.0

Coryphoideae, crown	66.0	51.35-80	0 = 0.716/1 = 0.28/2 = 0.0

Ceroxyloideae, crown	52.1	30-74.2	**0 = 0.979**/1 = 0.0/2 = 0.0

Arecoideae, crown	73.6	66.1-81.3	**0 = 0.999**/1 = 0.0/2 = 0.0

Bold entries indicate values significantly different from other states.

^a^0 = Tropical rain forest restricted; 1 = mangrove restricted; 2 = non-rain forest restricted.

HPD = highest posterior density; Ma = millions of years.

### Origin of tropical rain forests

The presence of TRF during the mid-Cretaceous period is controversial because pre-Cenozoic fossils associated with TRF are notoriously sparse [8,11,55,56] in contrast to the relatively well documented fossil evidence of TRF during the early Cenozoic from equatorial to relatively high palaeolatitudes [11-14,20,57]. The earliest fossil flora interpreted as characteristic of TRF was found in the early Cenomanian from several formations in North America, for example, the Dakota formation of Kansas (99 Ma, [9,53,58]), which is consistent with our results. However, these conclusions, which were based on the physiognomy of leaf characters, such as shape and size, that are generally associated with megathermal vegetation [20,53], have been questioned by some authors (see personal communication from Johnson in Morley [20]). Several studies have suggested that during the Cretaceous plant biodiversity was highest at mid paleolatitudes where the climate was more favourable while equatorial latitudes were exposed to a drier and hotter climate unlikely to have supported TRFs [20,59]. In addition, simulations of major vegetation distributions during the mid-Cretaceous period (100 Ma) indicate that the presence of TRF in the Cenomanian of North America and Eurasia as well as other parts of the world is plausible [14,60]. Finally, indirect evidence is provided by a diversification study of Malpighiales, a large plant order mainly restricted to TRFs. Using a molecular clock approach, it was estimated that the origin of this order dates to around 114 Ma with subsequent diversification during the Cenomanian [10], implying the presence of TRF at that time.

Even though molecular dating methods have been criticised in relation to interpretations of TRF origins [12] and are not assumption free, such approaches have played important roles in understanding the construction of other species rich biomes (for example, the Cape flora [61,62]) and provide important insights when the fossil record is sparse or incomplete. Molecular dating of palms, one of the most characteristic TRF plant families, provides additional evidence that modern TRF might have already been in place 100 Ma, significantly earlier than suggested by unequivocal fossil evidence [13,57]. It is most likely that formation of the TRF biome was a gradual process and thus the precise time at which modern TRFs can be recognised may be impossible to pinpoint. However, our results and other evidence discussed above [9,10,53] imply that the assembly of the TRF biome had already started during the mid-Cretaceous period and was not just a strictly Cenozoic process. It is puzzling that, to our knowledge, no macrofossil of palms has been recovered in Cenomanian deposits of North America [9]. However, our results suggest that palms were just starting to diversify at this time and may not have been widespread, thus reducing the frequency of fossilization and probability of later discovery. Interestingly, studies of other species-rich TRF plant families yield timings for the earliest extant lineage diversification events (that is, crown node estimates) that largely post-date palms, for example Leguminosae (59 Ma, [63]), Annonaceae (89 Ma, [64]) and Rubiaceae (86 Ma, [65]). This would imply that palms represent one of the first extant plant families to have diversified within TRF since its origin. Thus, palms not only play a major role in present day TRF [28-30], but also appear to have been a key component in the assembly and diversity of this biome since the earliest stages of its evolution.

### Early diversification of tropical rain forests

To depict global diversification at the family level and test the hypotheses of TRF diversification, we generated a semilogarithmic lineage-through-time (LTT) plot as well as 95% confidence intervals based on 1,000 randomly selected posterior trees from the BEAST output (Figure 3c). LTT plots are widely used to characterise the diversification of clades as a function of time [66-68], but they are sensitive to incomplete taxon sampling [69,70], as is the case here (183 species sampled out of circa 2,400, approximately 7.6%, see Additional file 1). However, our sampling is phylogenetically representative and non-random as we included 100% of all described palm genera. Such a sampling strategy has the advantage of representing all the deeper nodes of the phylogeny and can provide a good estimation of diversification history up to a point in time after which undersampling at shallower nodes biases the inference [71,72]. In order to restrict the interpretation of our LTT plot to the more accurately estimated parts we used a novel approach to identify a threshold after which the potential impact of incomplete taxon sampling becomes too important to permit accurate analyses of diversification rates (see Methods). Based on the chronogram, the percentage of total missing palm species increased sharply from 12% to 28% after circa 24 Ma (Figure 3c). Interpretation of the LTT plot was thus restricted to the period prior to that point in time (from 100 Ma to 24 Ma) and all nodes occurring after the 24 Ma threshold were excluded from subsequent analyses.

Between 100 Ma and 24 Ma, the LTT plot forms an almost straight line, which suggests that the palm family underwent a constant rate of diversification without any major shifts or radiations [67,68]. This is also statistically supported by the better fit of the pure-birth model of diversification (constant diversification rates with no extinction) on the LTT plot than any other model tested (ΔAIC_RC _= -1.421 between the pure birth model and the second best fitting model (the density-dependent or 'DDX' model); AIC = Akaike Information Criterion). Moreover, the ΔAIC_RC _was not significantly different under the null hypothesis of diversification rate consistency when calculated from 10,000 phylogenies simulated under the pure-birth process (*P *value = 0.739). Finally, the γ statistic [70] also supported a constant rate hypothesis as it was not significantly different from zero (γ = -0.318, *P *value = 0.379). These results lend support to the museum model (Figure 1) in which diversification rates remain constant and extinction rates are low [16,22]. This hypothesis was thought to be the consequence of the old and ecologically stable conditions of TRF over millions of years. Even though such views have now been replaced by the notion of greater dynamism in TRF evolution [14,17,73], the biome itself has never completely disappeared [14,20] and has persisted in refuges during unfavourable climatic times. The existence of such refuges may have allowed comparatively old lineages to persist and contribute to present day species diversity. For example, lineages of another diverse TRF plant family, Annonaceae, were shown to have persisted in possible TRF refuges of East and West Africa for over 30 million years [74] even during climatically unfavourable times (for example, the global cooling of the Eocene/Oligocene boundary). We suggest here that TRF refugia may have played a similar role throughout the history of the palm family and, as a result, global palm species diversity is at least partly the result of a gradual accumulation of ancestral lineages through time, and cannot be attributed to ancient or recent speciation bursts alone.

At finer time scales, palm species diversity and diversification rates most likely fluctuated with extinction rates possibly increasing and decreasing at specific time frames in the past, perhaps in relation to climatic and geological changes. For example, studies of the palynoflora through the Late Paleocene-Eocene Thermal Maximum in Colombia indicate an increase in palm morphospecies after this time (56.3 Ma, [8]). Thus, it is probable that different clades within palms underwent alternative diversification scenarios leading to a heterogeneous pattern of diversification among the lineages within the family. However, our study implies that over a larger time scale these changes did not influence the overall global pattern of diversification in palms, at least until 24 Ma. This is also apparent during the major extinction episode at the Cretaceous/Paleogene boundary (K/Pg; [75]), which had no statistically significant effect on diversification rates (Figure 3c).

To date, relatively few family-level studies have provided evidence for the museum model of tropical plant diversification (Annonaceae [64], liverwort family Lejeunaceae [76]). This pattern contrasts with the study of Malpighiales evolution, which indicated that all major lineages originated within a short timeframe suggesting an early rapid speciation of the order, although no detailed diversification analysis was undertaken for this group [10]. Interestingly, meta-analyses based on a large number of species-level dated molecular phylogenies of a range of plants and animals have also underlined the importance of the constant rate diversification model [77,78]. Indeed a large number of phylogenies fitted the simplest model of diversification. For example, Morlon *et al*. [78], using a novel coalescent-based approach, found that 87 out of 289 phylogenies studied (30%) better fitted the Yule process (time constant rates with no extinction) than alternative models. Even though these results were obtained from a wide range of organisms occurring in different ecosystems, it nevertheless underlines the importance of such a simple process for explaining present day diversity.

Given the threshold of 24 Ma imposed on our LTT plot (Figure 3c), there is little indication about recent (Neogene) diversification patterns. However, it is clear that in order to attain present day species diversity (Figure 3c), rates must have increased, which suggests a shift in diversification that occurred after 24 Ma. This could have been achieved either by accelerating diversification rates, consistent with the 'recent cradle' model, or simply by higher overall constant rates. Recent high speciation rates within other TRF plant genera have been documented [13-15] and it seems likely that rapid speciation occurred within some species-rich palm genera given the very young stem node age estimations we inferred for them (for example, *Pinanga*, approximately 130 species, stem node approximately 12 Ma; *Dypsis*, approximately 140 species, stem node approximately 13 Ma). In fact, the 'museum' and 'recent cradle' hypotheses are not mutually exclusive; both mechanisms could be at work, possibly within different palm lineages and different time frames. For example, both the 'ancient' and 'recent' cradle models of diversification have been identified within the TRF-restricted leaf beetle family [79].

## Conclusions

Our results from one of the most important TRF plant families suggests that present day TRF biodiversity can at least in part be explained by a steady accumulation of lineages dating back to the mid-Cretaceous period and is not just the result of rapid radiations, either recent [25] or ancient [10]. The analysis of additional family-level diversification patterns of other TRF restricted plant groups will undoubtedly shed more light into the evolutionary forces that have led to the immense diversity of species found within modern TRF today.

## Methods

### Taxon sampling

This study builds upon the complete generic-level supertree analyses of palms by Baker *et al*. [47], the most extensive phylogenetic study of the family published to date. Here, the sampling is updated to be consistent with the latest family-wide monograph [28,80], in which 183 genera are accepted (see Additional file 1 for the list of genera used). This was performed by repeating the supertree analyses of Baker *et al*. [47] with the addition of published plastid DNA sequence data for the recently described genus *Tahina *[81] (see below). Three commelinid monocot outgroup taxa (*Costus*, *Dasypogon*, *Zea*) were selected from the sampling of Baker *et al*. [47].

### Fossil calibration

Palms have a rich fossil record dating from the Late Cretaceous onwards. Although the record is unusually rich among angiosperms, only a small fraction of palm fossils can be identified to specific taxonomic groups with confidence. Drawing on recent surveys of the palm fossil record [28,82,83], we selected the most reliable fossils (Table 2), judged on the basis of the credibility of their purported taxonomic affinities and reported ages. Nevertheless, none of these fossils is sufficiently informative to justify allocation to crown nodes [84]; they are thus applied conservatively to stem nodes throughout. Where authors provide a range of age estimates, we have used the most recent date. Where a geological time period alone is specified, we have used the date of the upper end of that period [85].

**Table 2:** Names of fossils used to calibrate the tree, with the respective exponential prior parameters used

Fossil name	Hard lower bound (Ma)	Soft upper bound 95% (Ma)	Exponential mean (uncertainty)
Sabalites carolinensis	85.8	88.8	1

Mauritiidites	65	69.49	1.5

Attaleinae	54.8	60.79	2

Hyphaene kapelmanii	27	28.5	0.5

Ma = millions of years.

The four selected fossils are widely distributed across the family and are located in three out of the five subfamilies. The earliest fossils that can be assigned unequivocally to a taxonomic group within palms are Late Cretaceous records of palmate leaves, the earliest of which is *Sabalites carolinensis *from the late Coniacian of South Carolina [86]. Although an affinity with *Sabal *is implied by the genus name, the fossil could be linked with many coryphoid groups and its age is therefore used conservatively here as a calibration point for the stem node of subfamily Coryphoideae as a whole with an age of 85.8 Ma. *Hyphaene kapelmanii*, a fossil discovered at a late Oligocene site in Ethiopia [87], provides a further calibration point within the Coryphoideae. This fossil consists of a petiole fragment with a close resemblance to the modern genus *Hyphaene *due to the morphology of its wide, recurved marginal spines. We use this fossil as a constraint for the stem node of subtribe Hyphaeninae with an age of 27 Ma.

In subfamily Calamoideae, the unique structure of the pollen of subtribe Mauritiinae corresponds closely to fossil pollen in the genus *Mauritiidites*, specifically the clavate monosulcate grains with each spine inset and a swollen foot layer below. *Mauritiidites *has been recorded as early as the Maastrichtian of Africa [88] with numerous records soon after in the Palaeocene onwards of South America [89]. We use it here as a calibration for the stem node of the Mauritiinae with an age of 65 Ma.

Fossil records of the coconut tribe Cocoseae, particularly of fossilised endocarps, are numerous [28]. Until recently, well documented records appeared from the Middle Eocene onwards (for example, [90,91]), but new research in the middle to late Palaeocene of Colombia has revealed compression fossils of large fruits that closely resemble the modern coconut, *Cocos nucifera*, both in size and surface morphology [92]. In the absence of further substantiating evidence, we allocate this fossil to the stem node of the Attaleinae, the subtribe of tribe Cocoseae to which *Cocos *belongs, with an age of 54.8 Ma.

Finally, a number of other reliable fossils could not be used because they are made redundant by older fossils assigned to more distal nodes. *Nypa *is most notable here, given its outstanding macrofossil and microfossil records dating back to the Maastrichtian [28,93]. Also significant are the distinctive diaperturate fossil pollen grains, usually referred to the form genus *Dicolpopollis*, which can be assigned with confidence to tribe Calameae of the Calamoideae [82,83,94-96]. The earliest records of this fossil palynomorph are from the Maastrichtian and its boundary with the early Palaeocene of Somalia and Borneo [88,96].

For each fossil we applied an exponential prior, the parameters of which are given in Table 2. Finally, the stem node of palms was constrained by a uniform prior ranging from 110 to 120 Ma. This corresponds to the earliest monocot fossil [97]. By doing this we imply that the stem of palms cannot be older than the oldest monocot fossil.

### Molecular dating

Molecular dating was carried out using BEAST 1.5.3 [48,98]. For this analysis the 'most congruent supertree', based on the method and data of Baker *et al*. [47], was used as a topological constraint. This topology was based on extensive data sampling, including DNA sequence data, restriction fragment length polymorphisms (RFLP) and morphology, and represents the best family-wide estimation of phylogenetic relationships between palm genera to date. To update the taxonomic sampling of the supertree, we repeated the supertree analysis of Baker *et al*. [47]. This study used a matrix representation with parsimony (MRP) analysis based on input trees generated from individual partitions and combinations of partitions with matrix elements weighted in proportion to bootstrap values of corresponding input tree clades. The strict consensus tree of this analysis was highly resolved with minor ambiguity in tribe Trachycarpeae and parts of tribe Areceae only. One most-parsimonious tree was selected at random and used as a constraint. This tree was pruned to include only the 183 genera accepted by Dransfield *et al*. [28]. All DNA sequence datasets utilised by Baker *et al*. [47] were included in our molecular dating analysis (plastid DNA regions: *atpB*, *matK*, *ndhF*, *rbcL*, *rps16 *intron, *trnD*-*trnT*, *trnL*-*trnF*, *trnQ*-*rps16*; nuclear DNA regions: *18S*, *ITS*, *ms*, *prk*, *rpb2*). The completeness of taxonomic sampling for each of these regions varies from 12% to 100%, with an average of 48%. Moreover, sampling for the chloroplast markers was much more complete than for the nuclear markers. Morphological and RFLP datasets used by Baker *et al*. [47] were excluded. The supertree topology was used as constraint by deleting in the XLM BEAST input file the following commands: subtreeSlide; narrowExchange; wideExchange; wilsonBalding. Each marker was individually partitioned in BEAUTi 1.5.3 http://beast.bio.ed.ac.uk/BEAUti and assigned the General Time Reversible model (GTR) with gamma-distributed rate variation (G). model of sequence evolution. Prior to our full analysis, we investigated the effect of missing data on the estimation of ages. We undertook a preliminary analysis on 2 datasets: 1 with all the 13 markers (missing data present) and 1 where the 5 nuclear markers were removed (missing data of 10%). A regression analysis between the ages obtained for all nodes was highly significantly positive (R = 0.88; t test: *P *< 0.001) indicating that missing data in our dataset are likely to have little influence on age estimations in molecular dating. We then undertook the full-scale analysis on the full 13-marker dataset.

In total, 8 individual analyses were carried out, 5 with 20 million generations and 2 with 30 million generations, resulting in a total of 160 million generations, and sampling every 1,000th generation. Individual analyses were performed in order to test for convergence of the results. Tracer 1.4 [99] was used to check for convergence of the model likelihood and parameters between each run. Results were considered reliable once the effective sampling sizes (ESS) of all parameters exceeded 200. The resulting independent log and tree files were then combined using LogCombiner discarding 10% of generations as burn in per independent run.

Finally, deviation from a strict molecular clock was tested by running the analysis a second time with the strict clock enforced. We used the Bayes Factor as implemented in Tracer 1.4 [99] to select the best-fitting model under the smoothed marginal likelihood estimate and with 100 bootstrap replicates [100]. This test strongly supported the data as being non-clock like (ln BF = 1,158.2 ± 3.2 in favour of relaxed clock hypothesis), and thus the results under the relaxed clock are presented here.

### Diversification analyses

To explore diversification rates in the family, we generated a semilogarithmic LTT plot. The mean LTT plot as well as the 95% confidence intervals was generated from a random selection of 1,000 posterior trees resulting from the BEAST analysis. LTT plots are sensitive to incomplete taxon sampling [70]. However, the full generic sampling of palm genera (100% of genera included) means that sampling of extant lineages is complete in the older parts of the phylogeny, becoming progressively more incomplete towards the present [71,101]. In order to avoid misinterpretation of the plot we restrict our analyses to the accurately estimated part of the LTT, which will be least influenced by the missing taxa. We do this by finding the point at which incomplete taxon sampling will likely begin to have a significant effect on the LTT plot. Under the assumption that all genera are monophyletic, speciation events within each genus will always be found after the stem node of that genus. Given the known age of each stem node for each genus, we calculated a cumulative total for the number of missing species as a function of time. By doing this we generated a time-dependent curve representing the increase of missing taxa from the origin of the family until the present that quantified the amount of uncertainty each part of the LTT plot contains (Figure 3c). In this study, the proportion of missing taxa was less than 12% from 100 Ma to 24 Ma, at which time a dramatic increase occurred with missing species rising to 24% and on to > 92% at the present time. This point represents the stem age of the genus *Calamus*, the most species-rich genus in palms [28]. All nodes occurring after this 24 Ma threshold were deleted from subsequent diversification analyses.

Two different approaches were used to test for significant changes in diversification rates. First, we used a maximum likelihood method for fitting alternative diversification models to the LTT plot [102] using the R package LASER 2.2 [103]. The test statistic for diversification rate constancy ΔAIC_RC _is calculated as ΔAIC_RC _= AIC_RC _- AIC_Rv_, where AIC_Rc _is the AIC score for the best fitting rate-constant diversification model, and AIC_Rv _is the AIC for the best fitting variable-rate diversification model. A negative value for ΔAIC_RC _indicates that the data is best approximated by a rate-constancy model. We fitted five different diversification models: (1) the constant-rate birth model (the Yule process; [104]) with the speciation rate (λ) being constant and the extinction (μ) set to zero; (2) the constant-rate birth-death model with two parameters, speciation (λ) and extinction (μ); (3) a pure birth rate-variable model where the speciation rate λ1 shifts to rate λ2 at time ts, with three parameters (λ1, λ2, ts); (4) an exponential density-dependent speciation rate 'DDX' model; and (5) a logistic density-dependent speciation rate 'DDL' model. The significance of the observed ΔAIC_RC _was evaluated by simulating 10,000 trees under a pure birth constant diversification rate.

Second, we calculated the γ statistic of Pybus and Harvey [70], which provides a summary of the distribution of nodes in the phylogeny: if the internal nodes are closer to the root then γ < 0; if they are closer to the tips then γ > 0; if the nodes are equally spread out then γ = 0. The observed γ statistic was compared with the distribution of the γ statistic of 1,000 simulated phylogenies under a pure-birth model using the LASER package 2.2.

### Ancestral areas

A presence-absence matrix was built representing the global distribution of palm genera (see Additional file 1 for the original data used to perform this analysis). We defined seven, non-overlapping major palm areas that reflect the distribution and endemism of genera as well as broad scale geological units, as follows: (A) South America, (B) North America (including Central American and the Caribbean), (C) Africa (including Arabia); (D) Indian Ocean (Madagascar, Mascarenes, Comoros and Seychelles), (E) India (including Sri Lanka), (F) Eurasia (including west Malesia to the west of Wallace's Line) and (G) Pacific Ocean (including east Malesia to the east of Wallace's Line, Australia and the Pacific Islands). Each genus was assigned to one or more of the major palm areas based on its known current distribution [28,105].

Ancestral areas (AA) were reconstructed using a maximum likelihood method under the dispersal-extinction-cladogenesis model [49,50] as implemented in the software Lagrange build 20091004 [50]. We tested our ancestral area reconstruction under two different biogeographic models (see Additional file 1 for the parameters used to perform these analyses). The first model (M_0_) was unconstrained and we assigned an equal probability (*P *= 1.00) of dispersal between all areas during the whole time period considered. This model assumes that spatial relationships among areas have no effect on biogeographical patterns. For the second model (M_1_), we applied a more complex biogeographic scenario incorporating prior information on range evolution as well as dispersal probabilities between areas given discrete periods of time. This model was based on past climatic data, tectonic history and presence/absence of postulated land bridges [14,19,20,106-108]. Five time frames were delimited and dispersal probabilities were assigned between all adjacent areas (see Additional file 1). Dispersal probabilities were set as following: low or no dispersal = 0.01; low dispersal = 0.25; medium dispersal = 0.5; high dispersal = 0.75; areas adjacent or very close = 1. Scripts in the programming language Python http://www.python.org/ were generated using the online helper http://www.reelab.net/lagrange. Because of the large surface of each area (continent-level areas), the maximum number of ancestral areas was limited to two.

The genus *Cocos *presented a special problem because it is widely distributed across all areas. Such highly polymorphic states generally inject a high level of ambiguity into the analyses, as was the case in preliminary analyses here. Following the recommendations of Ronquist [109], we allocated a putative ancestral area coding to *Cocos *(area A) based on the findings of Meerow *et al*. [110].

### Ancestral biome

The probable ancestral biome at the crown node of palms was reconstructed under a maximum likelihood method using the Markov k-state 1 parameter model(Mk_1_) model of character evolution implemented in Mesquite version 2.74 [10,111]. We assigned genera into three different biome state categories following Olson *et al*. [112]. State 0: predominantly 'tropical and subtropical moist broadleaf forests' biome (that is, TRF); state 1: 'mangrove'; state 2: a general category that contains genera not belonging to any of the two first categories (that is, not TRF-restricted). State 2 is broad in its definition encompassing all other biomes for palms. When a genus occurred in both TRF and non-TRF biomes it was coded as ambiguous (see Additional file 1 for the original data used to perform this analysis). The mangrove category was included to take into account the ecology of *Nypa*. The phylogenetic signal of the biome character was tested by randomising the tips of the phylogeny 1,000 times in Mesquite [111] in order to create a null distribution of the number of steps under the maximum parsimony criterion and the 99% confidence intervals. This null distribution was compared to the observed number of steps necessary to explain the occurrence of each character on the phylogeny. In this case, the observed value fell outside the 99% confidence interval confirming that the biome category is phylogenetically conserved.

## Authors' contributions

TLPC and WJB designed the study; TLPC, FF and WJB performed the research, analysed the data and wrote the paper. All authors read and approved the final manuscript.

## Supplementary Material

Additional file 1 **Additional tables**. Table S1: Genera sampled with total number of species per genus, biome coding used in Mesquite and area coding used in the Lagrange analysis. Table S2: Alternative dispersal models between areas used in Lagrange. This file presents the names of all officially recognised palm genera, with the coding for present day biome and area, as well as the details of the two alternative biogeographical models used in the analysis. Click here for file
