# Supplementary material for: Origin and global diversification patterns of tropical rain forests: inferences from a complete genus-level phylogeny of palms
Source: BMC Biol. 2011 Jun 16;9:44. doi: 10.1186/1741-7007-9-44 (PMC3142250; doi:10.1186/1741-7007-9-44)
Supplement: Additional file 1 — Additional tables. Table S1: Genera sampled with total number of species per genus, biome coding used in Mesquite and area coding used in the Lagrange analysis. Table S2: Alternative dispersal models between areas used in Lagrange. This file presents the names of all officially recognised palm genera, with the coding for present day biome and area, as well as the details of the two alternative biogeographical models used in the analysis. [file 1741-7007-9-44-S1.DOC]

**Additional tables**

**Couvreur et al.**

Tables

|  |  |  |  | Ancestral areas | | | | | | |
| --- | --- | --- | --- | --- | --- | --- | --- | --- | --- | --- |
| **Genus** | **Number of species** | **Biome** |  | **A** | **B** | **C** | **D** | **E** | **F** | **G** |
| *Acanthophoenix* | 3 | 0 |  | 0 | 0 | 0 | **1** | 0 | 0 | 0 |
| *Acoelorraphe* | 1 | 1 |  | 0 | **1** | 0 | 0 | 0 | 0 | 0 |
| *Acrocomia* | 34 | 1 |  | **1** | **1** | 0 | 0 | 0 | 0 | 0 |
| *Actinokentia* | 2 | 0 |  | 0 | 0 | 0 | 0 | 0 | 0 | **1** |
| *Actinorhytis* | 1 | 0 |  | 0 | 0 | 0 | 0 | 0 | 0 | **1** |
| *Adonidia* | 1 | 0 |  | 0 | 0 | 0 | 0 | 0 | **1** | 0 |
| *Aiphanes* | 24 | 0 |  | **1** | **1** | 0 | 0 | 0 | 0 | 0 |
| *Allagoptera* | 5 | 1 |  | **1** | 0 | 0 | 0 | 0 | 0 | 0 |
| *Ammandra* | 1 | 0 |  | **1** | 0 | 0 | 0 | 0 | 0 | 0 |
| *Aphandra* | 1 | 0 |  | **1** | 0 | 0 | 0 | 0 | 0 | 0 |
| *Archontophoenix* | 6 | ? |  | 0 | 0 | 0 | 0 | 0 | 0 | **1** |
| *Areca* | 47 | 0 |  | 0 | 0 | 0 | 0 | **1** | **1** | **1** |
| *Arenga* | 25 | 0 |  | 0 | 0 | 0 | 0 | **1** | **1** | **1** |
| *Asterogyne* | 5 | 0 |  | **1** | **1** | 0 | 0 | 0 | 0 | 0 |
| *Astrocaryum* | 40 | 0 |  | **1** | **1** | 0 | 0 | 0 | 0 | 0 |
| *Attalea* | 69 | ? |  | **1** | **1** | 0 | 0 | 0 | 0 | 0 |
| *Bactris* | 77 | 0 |  | **1** | **1** | 0 | 0 | 0 | 0 | 0 |
| *Balaka* | 11 | 0 |  | 0 | 0 | 0 | 0 | 0 | 0 | **1** |
| *Barcella* | 1 | 0 |  | **1** | 0 | 0 | 0 | 0 | 0 | 0 |
| *Basselinia* | 12 | 0 |  | 0 | 0 | 0 | 0 | 0 | 0 | **1** |
| *Beccariophoenix* | 2 | 0 |  | 0 | 0 | 0 | **1** | 0 | 0 | 0 |
| *Bentinckia* | 2 | 0 |  | 0 | 0 | 0 | 0 | **1** | **1** | 0 |
| *Bismarckia* | 1 | 1 |  | 0 | 0 | 0 | **1** | 0 | 0 | 0 |
| *Borassodendron* | 2 | 0 |  | 0 | 0 | 0 | 0 | 0 | **1** | 0 |
| *Borassus* | 6 | ? |  | 0 | 0 | **1** | **1** | **1** | **1** | **1** |
| *Brahea* | 10 | 1 |  | 0 | **1** | 0 | 0 | 0 | 0 | 0 |
| *Brassiophoenix* | 1 | 0 |  | 0 | 0 | 0 | 0 | 0 | 0 | **1** |
| *Burretiokentia* | 5 | 0 |  | 0 | 0 | 0 | 0 | 0 | 0 | **1** |
| *Butia* | 9 | 1 |  | **1** | 0 | 0 | 0 | 0 | 0 | 0 |
| *Calamus* | 374 | 0 |  | 0 | 0 | **1** | 0 | **1** | **1** | **1** |
| *Calyptrocalyx* | 27 | 0 |  | 0 | 0 | 0 | 0 | 0 | 0 | **1** |
| *Calyptrogyne* | 9 | 0 |  | 0 | **1** | 0 | 0 | 0 | 0 | 0 |
| *Calyptronoma* | 3 | 0 |  | 0 | **1** | 0 | 0 | 0 | 0 | 0 |
| *Carpentaria* | 1 | 0 |  | 0 | 0 | 0 | 0 | 0 | 0 | **1** |
| *Carpoxylon* | 1 | 0 |  | 0 | 0 | 0 | 0 | 0 | 0 | **1** |
| *Caryota* | 13 | 0 |  | 0 | 0 | 0 | 0 | **1** | **1** | **1** |
| *Ceratolobus* | 6 | 0 |  | 0 | 0 | 0 | 0 | 0 | **1** | 0 |
| *Ceroxylon* | 11 | 0 |  | **1** | 0 | 0 | 0 | 0 | 0 | 0 |
| *Chamaedorea* | 110 | 0 |  | **1** | **1** | 0 | 0 | 0 | 0 | 0 |
| *Chamaerops* | 1 | 1 |  | 0 | 0 | **1** | 0 | 0 | **1** | 0 |
| *Chambeyronia* | 2 | 0 |  | 0 | 0 | 0 | 0 | 0 | 0 | **1** |
| *Chelyocarpus* | 4 | 0 |  | **1** | 0 | 0 | 0 | 0 | 0 | 0 |
| *Chuniophoenix* | 3 | 0 |  | 0 | 0 | 0 | 0 | 0 | **1** | 0 |
| *Clinosperma* | 4 | 0 |  | 0 | 0 | 0 | 0 | 0 | 0 | **1** |
| *Clinostigma* | 11 | 0 |  | 0 | 0 | 0 | 0 | 0 | 0 | **1** |
| *Coccothrinax* | 50 | 1 |  | 0 | **1** | 0 | 0 | 0 | 0 | 0 |
| *Cocos* | 1 | 0 |  | **1** | 0 | 0 | 0 | 0 | 0 | 0 |
| *Colpothrinax* | 3 | ? |  | 0 | **1** | 0 | 0 | 0 | 0 | 0 |
| *Copernicia* | 21 | 1 |  | **1** | **1** | 0 | 0 | 0 | 0 | 0 |
| *Corypha* | 6 | ? |  | 0 | 0 | 0 | 0 | **1** | **1** | **1** |
| *Cryosophila* | 10 | ? |  | 0 | **1** | 0 | 0 | 0 | 0 | 0 |
| *Cyphokentia* | 2 | 0 |  | 0 | 0 | 0 | 0 | 0 | 0 | **1** |
| *Cyphophoenix* | 4 | 0 |  | 0 | 0 | 0 | 0 | 0 | 0 | **1** |
| *Cyphosperma* | 4 | 0 |  | 0 | 0 | 0 | 0 | 0 | 0 | **1** |
| *Cyrtostachys* | 7 | 0 |  | 0 | 0 | 0 | 0 | 0 | **1** | **1** |
| *Daemonorops* | 101 | 0 |  | 0 | 0 | 0 | 0 | **1** | **1** | **1** |
| *Deckenia* | 1 | 0 |  | 0 | 0 | 0 | **1** | 0 | 0 | 0 |
| *Desmoncus* | 23 | 0 |  | **1** | **1** | 0 | 0 | 0 | 0 | 0 |
| *Dictyocaryum* | 3 | 0 |  | **1** | 0 | 0 | 0 | 0 | 0 | 0 |
| *Dictyosperma* | 1 | 0 |  | 0 | 0 | 0 | **1** | 0 | 0 | 0 |
| *Dransfieldia* | 1 | 0 |  | 0 | 0 | 0 | 0 | 0 | 0 | **1** |
| *Drymophloeus* | 8 | 0 |  | 0 | 0 | 0 | 0 | 0 | 0 | **1** |
| *Dypsis* | 140 | 0 |  | 0 | 0 | 0 | **1** | 0 | 0 | 0 |
| *Elaeis* | 2 | 0 |  | **1** | **1** | **1** | 0 | 0 | 0 | 0 |
| *Eleiodoxa* | 1 | 0 |  | 0 | 0 | 0 | 0 | 0 | **1** | 0 |
| *Eremospatha* | 10 | 0 |  | 0 | 0 | **1** | 0 | 0 | 0 | 0 |
| *Eugeissona* | 6 | 0 |  | 0 | 0 | 0 | 0 | 0 | **1** | 0 |
| *Euterpe* | 7 | 0 |  | **1** | **1** | 0 | 0 | 0 | 0 | 0 |
| *Gaussia* | 5 | 1 |  | 0 | **1** | 0 | 0 | 0 | 0 | 0 |
| *Geonoma* | 68 | 0 |  | **1** | **1** | 0 | 0 | 0 | 0 | 0 |
| *Guihaia* | 2 | 0 |  | 0 | 0 | 0 | 0 | 0 | **1** | 0 |
| *Hedyscepe* | 1 | 0 |  | 0 | 0 | 0 | 0 | 0 | 0 | **1** |
| *Hemithrinax* | 3 | 1 |  | 0 | **1** | 0 | 0 | 0 | 0 | 0 |
| *Heterospathe* | 40 | 0 |  | 0 | 0 | 0 | 0 | 0 | **1** | **1** |
| *Howea* | 2 | 0 |  | 0 | 0 | 0 | 0 | 0 | 0 | **1** |
| *Hydriastele* | 47 | 0 |  | 0 | 0 | 0 | 0 | 0 | 0 | **1** |
| *Hyophorbe* | 5 | 0 |  | 0 | 0 | 0 | **1** | 0 | 0 | 0 |
| *Hyospathe* | 6 | 0 |  | **1** | **1** | 0 | 0 | 0 | 0 | 0 |
| *Hyphaene* | 8 | 1 |  | 0 | 0 | **1** | **1** | **1** | 0 | 0 |
| *Iguanura* | 32 | 0 |  | 0 | 0 | 0 | 0 | 0 | **1** | 0 |
| *Iriartea* | 1 | 0 |  | **1** | **1** | 0 | 0 | 0 | 0 | 0 |
| *Iriartella* | 2 | 0 |  | **1** | 0 | 0 | 0 | 0 | 0 | 0 |
| *Itaya* | 1 | 0 |  | **1** | 0 | 0 | 0 | 0 | 0 | 0 |
| *Johannesteijsmannia* | 4 | 0 |  | 0 | 0 | 0 | 0 | 0 | **1** | 0 |
| *Juania* | 1 | 0 |  | 0 | 0 | 0 | 0 | 0 | 0 | **1** |
| *Jubaea* | 1 | 1 |  | **1** | 0 | 0 | 0 | 0 | 0 | 0 |
| *Jubaeopsis* | 1 | 0 |  | 0 | 0 | **1** | 0 | 0 | 0 | 0 |
| *Kentiopsis* | 4 | 0 |  | 0 | 0 | 0 | 0 | 0 | 0 | **1** |
| *Kerriodoxa* | 1 | 0 |  | 0 | 0 | 0 | 0 | 0 | **1** | 0 |
| *Korthalsia* | 26 | 0 |  | 0 | 0 | 0 | 0 | 0 | **1** | **1** |
| *Laccospadix* | 1 | 0 |  | 0 | 0 | 0 | 0 | 0 | 0 | **1** |
| *Laccosperma* | 5 | 0 |  | 0 | 0 | **1** | 0 | 0 | 0 | 0 |
| *Latania* | 3 | 1 |  | 0 | 0 | 0 | **1** | 0 | 0 | 0 |
| *Lemurophoenix* | 1 | 0 |  | 0 | 0 | 0 | **1** | 0 | 0 | 0 |
| *Leopoldinia* | 3 | 0 |  | **1** | 0 | 0 | 0 | 0 | 0 | 0 |
| *Lepidocaryum* | 1 | 0 |  | **1** | 0 | 0 | 0 | 0 | 0 | 0 |
| *Lepidorrhachis* | 1 | 0 |  | 0 | 0 | 0 | 0 | 0 | 0 | **1** |
| *Leucothrinax* | 1 | 1 |  | 0 | **1** | 0 | 0 | 0 | 0 | 0 |
| *Licuala* | 134 | 0 |  | 0 | 0 | 0 | 0 | **1** | **1** | **1** |
| *Linospadix* | 9 | 0 |  | 0 | 0 | 0 | 0 | 0 | 0 | **1** |
| *Livistona* | 35 | ? |  | 0 | 0 | **1** | 0 | **1** | **1** | **1** |
| *Lodoicea* | 1 | 0 |  | 0 | 0 | 0 | **1** | 0 | 0 | 0 |
| *Loxococcus* | 1 | 0 |  | 0 | 0 | 0 | 0 | **1** | 0 | 0 |
| *Lytocaryum* | 2 | 0 |  | **1** | 0 | 0 | 0 | 0 | 0 | 0 |
| *Manicaria* | 1 | 0 |  | **1** | **1** | 0 | 0 | 0 | 0 | 0 |
| *Marojejya* | 2 | 0 |  | 0 | 0 | 0 | **1** | 0 | 0 | 0 |
| *Masoala* | 2 | 0 |  | 0 | 0 | 0 | **1** | 0 | 0 | 0 |
| *Mauritia* | 2 | ? |  | **1** | 0 | 0 | 0 | 0 | 0 | 0 |
| *Mauritiella* | 3 | 0 |  | **1** | 0 | 0 | 0 | 0 | 0 | 0 |
| *Maxburretia* | 3 | ? |  | 0 | 0 | 0 | 0 | 0 | **1** | 0 |
| *Medemia* | 2 | 1 |  | 0 | 0 | **1** | 0 | 0 | 0 | 0 |
| *Metroxylon* | 7 | 0 |  | 0 | 0 | 0 | 0 | 0 | 0 | **1** |
| *Myrialepis* | 1 | 0 |  | 0 | 0 | 0 | 0 | 0 | **1** | 0 |
| *Nannorrhops* | 1 | 1 |  | 0 | 0 | **1** | 0 | 0 | **1** | 0 |
| *Nenga* | 5 | 0 |  | 0 | 0 | 0 | 0 | 0 | **1** | 0 |
| *Neonicholsonia* | 1 | 0 |  | 0 | **1** | 0 | 0 | 0 | 0 | 0 |
| *Neoveitchia* | 2 | 0 |  | 0 | 0 | 0 | 0 | 0 | 0 | **1** |
| *Nephrosperma* | 1 | 0 |  | 0 | 0 | 0 | **1** | 0 | 0 | 0 |
| *Normanbya* | 1 | 0 |  | 0 | 0 | 0 | 0 | 0 | 0 | **1** |
| *Nypa* | 1 | 2 |  | 0 | 0 | 0 | 0 | **1** | **1** | **1** |
| *Oenocarpus* | 9 | 0 |  | **1** | **1** | 0 | 0 | 0 | 0 | 0 |
| *Oncocalamus* | 5 | 0 |  | 0 | 0 | **1** | 0 | 0 | 0 | 0 |
| *Oncosperma* | 5 | 0 |  | 0 | 0 | 0 | 0 | **1** | **1** | **1** |
| *Orania* | 25 | 0 |  | 0 | 0 | 0 | **1** | 0 | **1** | **1** |
| *Oraniopsis* | 1 | 0 |  | 0 | 0 | 0 | 0 | 0 | 0 | **1** |
| *Parajubaea* | 3 | 1 |  | **1** | 0 | 0 | 0 | 0 | 0 | 0 |
| *Pelagodoxa* | 1 | 0 |  | 0 | 0 | 0 | 0 | 0 | 0 | **1** |
| *Phoenicophorium* | 1 | 0 |  | 0 | 0 | 0 | **1** | 0 | 0 | 0 |
| *Phoenix* | 14 | ? |  | 0 | 0 | **1** | **1** | **1** | **1** | 0 |
| *Pholidocarpus* | 6 | 0 |  | 0 | 0 | 0 | 0 | 0 | **1** | **1** |
| *Pholidostachys* | 4 | 0 |  | **1** | **1** | 0 | 0 | 0 | 0 | 0 |
| *Physokentia* | 7 | 0 |  | 0 | 0 | 0 | 0 | 0 | 0 | **1** |
| *Phytelephas* | 6 | 0 |  | **1** | 0 | 0 | 0 | 0 | 0 | 0 |
| *Pigafetta* | 2 | 0 |  | 0 | 0 | 0 | 0 | 0 | 0 | **1** |
| *Pinanga* | 131 | 0 |  | 0 | 0 | 0 | 0 | **1** | **1** | **1** |
| *Plectocomia* | 16 | 0 |  | 0 | 0 | 0 | 0 | **1** | **1** | 0 |
| *Plectocomiopsis* | 5 | 0 |  | 0 | 0 | 0 | 0 | 0 | **1** | 0 |
| *Podococcus* | 2 | 0 |  | 0 | 0 | **1** | 0 | 0 | 0 | 0 |
| *Pogonotium* | 3 | 0 |  | 0 | 0 | 0 | 0 | 0 | **1** | 0 |
| *Ponapea* | 3 | 0 |  | 0 | 0 | 0 | 0 | 0 | 0 | **1** |
| *Prestoea* | 10 | 0 |  | **1** | **1** | 0 | 0 | 0 | 0 | 0 |
| *Pritchardia* | 27 | 0 |  | 0 | 0 | 0 | 0 | 0 | 0 | **1** |
| *Pseudophoenix* | 4 | 1 |  | 0 | **1** | 0 | 0 | 0 | 0 | 0 |
| *Ptychococcus* | 2 | 0 |  | 0 | 0 | 0 | 0 | 0 | 0 | **1** |
| *Ptychosperma* | 29 | 0 |  | 0 | 0 | 0 | 0 | 0 | 0 | **1** |
| *Raphia* | 20 | 0 |  | **1** | **1** | **1** | 0 | 0 | 0 | 0 |
| *Ravenea* | 18 | 0 |  | 0 | 0 | 0 | **1** | 0 | 0 | 0 |
| *Reinhardtia* | 6 | 0 |  | 0 | **1** | 0 | 0 | 0 | 0 | 0 |
| *Retispatha* | 1 | 0 |  | 0 | 0 | 0 | 0 | 0 | **1** | 0 |
| *Rhapidophyllum* | 1 | 1 |  | 0 | **1** | 0 | 0 | 0 | 0 | 0 |
| *Rhapis* | 8 | 0 |  | 0 | 0 | 0 | 0 | 0 | **1** | 0 |
| *Rhopaloblaste* | 6 | 0 |  | 0 | 0 | 0 | 0 | 0 | **1** | **1** |
| *Rhopalostylis* | 2 | 1 |  | 0 | 0 | 0 | 0 | 0 | 0 | **1** |
| *Roscheria* | 1 | 0 |  | 0 | 0 | 0 | **1** | 0 | 0 | 0 |
| *Roystonea* | 10 | ? |  | **1** | **1** | 0 | 0 | 0 | 0 | 0 |
| *Sabal* | 16 | 1 |  | **1** | **1** | 0 | 0 | 0 | 0 | 0 |
| *Salacca* | 20 | 0 |  | 0 | 0 | 0 | 0 | 0 | **1** | 0 |
| *Saribus* | 1 | 0 |  | 0 | 0 | 0 | 0 | 0 | **1** | **1** |
| *Satakentia* | 1 | 0 |  | 0 | 0 | 0 | 0 | 0 | **1** | 0 |
| *Satranala* | 1 | 0 |  | 0 | 0 | 0 | **1** | 0 | 0 | 0 |
| *Schippia* | 1 | ? |  | 0 | **1** | 0 | 0 | 0 | 0 | 0 |
| *Sclerosperma* | 3 | 0 |  | 0 | 0 | **1** | 0 | 0 | 0 | 0 |
| *Serenoa* | 1 | 1 |  | 0 | **1** | 0 | 0 | 0 | 0 | 0 |
| *Socratea* | 5 | 0 |  | **1** | **1** | 0 | 0 | 0 | 0 | 0 |
| *Solfia* | 1 | 0 |  | 0 | 0 | 0 | 0 | 0 | 0 | **1** |
| *Sommieria* | 1 | 0 |  | 0 | 0 | 0 | 0 | 0 | 0 | **1** |
| *Syagrus* | 31 | ? |  | **1** | **1** | 0 | 0 | 0 | 0 | 0 |
| *Synechanthus* | 2 | 0 |  | **1** | **1** | 0 | 0 | 0 | 0 | 0 |
| *Tahina* | 1 | 1 |  | 0 | 0 | 0 | **1** | 0 | 0 | 0 |
| *Tectiphiala* | 1 | 0 |  | 0 | 0 | 0 | **1** | 0 | 0 | 0 |
| *Thrinax* | 3 | ? |  | 0 | **1** | 0 | 0 | 0 | 0 | 0 |
| *Trachycarpus* | 9 | ? |  | 0 | 0 | 0 | 0 | 0 | **1** | 0 |
| *Trithrinax* | 3 | 1 |  | **1** | 0 | 0 | 0 | 0 | 0 | 0 |
| *Veitchia* | 8 | 0 |  | 0 | 0 | 0 | 0 | 0 | 0 | **1** |
| *Verschaffeltia* | 1 | 0 |  | 0 | 0 | 0 | **1** | 0 | 0 | 0 |
| *Voanioala* | 1 | 0 |  | 0 | 0 | 0 | **1** | 0 | 0 | 0 |
| *Wallichia* | 9 | 0 |  | 0 | 0 | 0 | 0 | **1** | **1** | 0 |
| *Washingtonia* | 2 | 1 |  | 0 | **1** | 0 | 0 | 0 | 0 | 0 |
| *Welfia* | 1 | 0 |  | **1** | **1** | 0 | 0 | 0 | 0 | 0 |
| *Wendlandiella* | 1 | 0 |  | **1** | 0 | 0 | 0 | 0 | 0 | 0 |
| *Wettinia* | 21 | 0 |  | **1** | **1** | 0 | 0 | 0 | 0 | 0 |
| *Wodyetia* | 1 | 0 |  | 0 | 0 | 0 | 0 | 0 | 0 | **1** |
| *Zombia* | 1 | 0 |  | 0 | **1** | 0 | 0 | 0 | 0 | 0 |

Table S1: Genera sampled with total number of species per genus, biome coding used in Mesquite and area coding used in the Lagrange analysis. Data were obtained from ^1^

A) South America, B) North America (including Central American and the Caribbean), C) Africa (including Arabia); D) Indian Ocean (Madagascar, Mascarenes, Comoros and Seychelles), E) India (including Sri Lanka), F) Eurasia (including west Malesia to the west of Wallace’s Line) and G) Pacific Ocean (including east Malesia to the east of Wallace’s Line, Australia and the Pacific Islands).

0: Predominantly “Tropical and Subtropical Moist Broadleaf Forests” biome (i.e. TRF); 1: “Mangrove”; 2: a general category that contains genera not belonging to any of the two first categories (i.e. not rain forests restricted).

^1^ Dransfield, J., N. W. Uhl, C. B. Asmussen, W. J. Baker, M. M. Harley, and C. E. Lewis. 2008. Genera Palmarum - The Evolution and Classification of Palms. Royal Botanic Gardens, Kew.

|  | **Model 0: Unconstrained** | | | | | | |  |  | **Model 1: Constrained** | | | | | | |  |
| --- | --- | --- | --- | --- | --- | --- | --- | --- | --- | --- | --- | --- | --- | --- | --- | --- | --- |
| **0-100 Ma** | A | B | C | D | E | F | G |  | **0-5 Ma** | A | B | C | D | E | F | G | |
| A |  | 1 | 1 | 1 | 1 | 1 | 1 |  | A |  | 1 | 0.01 | 0.01 | 0.01 | 0.01 | 0.01 | |
| B |  |  | 1 | 1 | 1 | 1 | 1 |  | B |  |  | 0.01 | 0.01 | 0.01 | 0.01 | 0.01 | |
| C |  |  |  | 1 | 1 | 1 | 1 |  | C |  |  |  | 0.25 | 0.25 | 0.25 | 0.01 | |
| D |  |  |  |  | 1 | 1 | 1 |  | D |  |  |  |  | 0.25 | 0.25 | 0.01 | |
| E |  |  |  |  |  | 1 | 1 |  | E |  |  |  |  |  | 1 | 0.01 | |
| F |  |  |  |  |  |  | 1 |  | F |  |  |  |  |  |  | 1 | |
|  |  |  |  |  |  |  |  |  | **5-30 Ma** | A | B | C | D | E | F | G | |
|  |  |  |  |  |  |  |  |  | A |  | 0.25 | 0.01 | 0.01 | 0.01 | 0.01 | 0.01 | |
|  |  |  |  |  |  |  |  |  | B |  |  | 0.01 | 0.01 | 0.01 | 0.01 | 0.01 | |
|  |  |  |  |  |  |  |  |  | C |  |  |  | 0.5 | 0.25 | 0.25 | 0.01 | |
|  |  |  |  |  |  |  |  |  | D |  |  |  |  | 0.25 | 0.25 | 0.01 | |
|  |  |  |  |  |  |  |  |  | E |  |  |  |  |  | 1 | 0.01 | |
|  |  |  |  |  |  |  |  |  | F |  |  |  |  |  |  | 0.75 | |
|  |  |  |  |  |  |  |  |  | **30-45 Ma** | A | B | C | D | E | F | G | |
|  |  |  |  |  |  |  |  |  | A |  | 0.01 | 0.25 | 0.01 | 0.01 | 0.01 | 0.01 | |
|  |  |  |  |  |  |  |  |  | B |  |  | 0.01 | 0.01 | 0.01 | 0.01 | 0.01 | |
|  |  |  |  |  |  |  |  |  | C |  |  |  | 0.5 | 0.01 | 0.25 | 0.01 | |
|  |  |  |  |  |  |  |  |  | D |  |  |  |  | 0.01 | 0.25 | 0.01 | |
|  |  |  |  |  |  |  |  |  | E |  |  |  |  |  | 0.75 | 0.25 | |
|  |  |  |  |  |  |  |  |  | F |  |  |  |  |  |  | 0.25 | |
|  |  |  |  |  |  |  |  |  | **45-65 Ma** | A | B | C | D | E | F | G | |
|  |  |  |  |  |  |  |  |  | A |  | 0.25 | 0.5 | 0.01 | 0.01 | 0.01 | 0.01 | |
|  |  |  |  |  |  |  |  |  | B |  |  | 0.75 | 0.01 | 0.01 | 0.75 | 0.01 | |
|  |  |  |  |  |  |  |  |  | C |  |  |  | 0.5 | 0.01 | 0.25 | 0.01 | |
|  |  |  |  |  |  |  |  |  | D |  |  |  |  | 0.01 | 0.25 | 0.01 | |
|  |  |  |  |  |  |  |  |  | E |  |  |  |  |  | 0.25 | 0.25 | |
|  |  |  |  |  |  |  |  |  | F |  |  |  |  |  |  | 0.01 | |
|  |  |  |  |  |  |  |  |  | **65-100 Ma** | A | B | C | D | E | F | G | |
|  |  |  |  |  |  |  |  |  | A |  | 0.5 | 1 | 0.01 | 0.01 | 0.01 | 0.75 | |
|  |  |  |  |  |  |  |  |  | B |  |  | 0.25 | 0.01 | 0.01 | 0.5 | 0.01 | |
|  |  |  |  |  |  |  |  |  | C |  |  |  | 1 | 1 | 0.01 | 0.5 | |
|  |  |  |  |  |  |  |  |  | D |  |  |  |  | 1 | 0.01 | 0.75 | |
|  |  |  |  |  |  |  |  |  | E |  |  |  |  |  | 0.01 | 0.75 | |
|  |  |  |  |  |  |  |  |  | F |  |  |  |  |  |  | 0.01 | |

Table S2. Alternative dispersal models between areas used in Lagrange. A) South America, B) North America (including Central American and the Caribbean), C) Africa (including Arabia); D) Indian Ocean (Madagascar, Mascarenes, Comoros and Seychelles), E) India (including Sri Lanka), F) Eurasia (including west Malesia to the west of Wallace’s Line) and G) Pacific Ocean (including east Malesia to the east of Wallace’s Line, Australia and the Pacific Islands). Ma: million of years ago. Values represent relative probability of dispersal between areas.
